# Bio-Based Coatings on Cellulosic Materials Resistant to Humidity and Fats

**DOI:** 10.3390/polym17202755

**Published:** 2025-10-15

**Authors:** Bastián Rozas, Julio E. Bruna, Abel Guarda, María José Galotto, Cristopher Reyes, Ximena Valenzuela, Francisco Rodríguez-Mercado, Alejandra Torres

**Affiliations:** 1 Packaging Innovation Center (LABEN), University of Santiago of Chile (USACH), Santiago 9170022, Chile; bastian.rozas@usach.cl (B.R.); francisco.rodriguez.m@usach.cl (F.R.-M.); alejandra.torresm@usach.cl (A.T.); 2 Center for the Development of Nanoscience and Nanotechnology (CEDENNA), University of Santiago of Chile (USACH), Santiago 9170022, Chile; 3 Food Science and Technology Department (DECYTAL), Technological Faculty, University of Santiago of Chile (USACH), Santiago 9170022, Chile

**Keywords:** packaging paperboard, cellulose, water resistance, grease resistance, food packaging

## Abstract

Cellulose stands out as a promising alternative to conventional polymers in food packaging due to its abundance, renewability, biodegradability and structural robustness. Despite these advantages, its natural low resistance to water and fats limits its direct application, necessitating the use of protective coatings to enhance its functionality. In this context, the use of biopolymeric coatings such as poly(lactic acid) (PLA), starch, lignin, chitin and chitosan has emerged as a sustainable solution, providing effective barriers against moisture and oils. These coatings not only improve the functional performance of cellulosic substrates but also reduce reliance on fossil-based plastics, fostering compostable systems and supporting a circular economy. This review analyzes recent developments in biopolymer-coated cellulosic packaging materials, focusing on their resistance to water and fats. The aim is to assess their potential for sustainable food packaging applications. The findings highlight how these innovations contribute to global sustainability goals, such as reducing plastic waste, lowering carbon emissions, and decreasing dependence on non-renewable resources.

## 1. Introduction

Nowadays, one of the most worrying issues in society is the environmental problem generated by the massive use of plastic of fossil origin, where the global plastic production is estimated around 400 million tons a year, accumulating a great part in land and marine ecosystems related to its low biodegradability, affecting biodiversity as well as contributing to chemical and physical pollution on lands and water resources [1,2,3].

A second great problem that causes significant impacts on society, economy and environment relies on food waste, where the UN Environmental Programme (UNEP) estimated that 1050 million tons were wasted in 2022, which represents 19% of available food for consumers [4]. This problem worsens due to the low capacity of many traditional plastic packaging to keep quality and safety of food under adverse transport and storage conditions.

In this respect, containers elaborated from cellulosic materials such as paper and cardboard have been seen as a promising alternative for food packaging because of its renewable, biodegradable and compostable nature [5]. Different from traditional plastic derived from fuel, these materials show a lower environmental impact during its life cycle, contributing to reducing the accumulation of plastic waste in the environment [6]. However, one of the main limitations of these containers relies on their deficient performance in acting as a protective barrier to gases, humidity and fats, which affects negatively its functionality as material for food packaging [7].

Currently, to address this problem, the treatment of these materials through the coatings of their layers to obtain a high resistance against water and oils is considered as an effective solution. Nevertheless, most of these products are used as compounds derived from fuel, which show difficulties in splitting from the cellulosic substrate, negatively impacting the environment, and therefore compromising its recyclization [8]. Likewise, current laws globally show an increasing orientation to the protection of the ecosystem and promotion of circular economy under several regulation frames which prioritize sustainability, efficient use of resources and extended producer responsibility, aiming at moving towards balanced development models and being respectful to the environment.

To face these challenges, various studies have focused on the development of coatings based on natural polymers derived from renewable biological sources, known as biopolymers, with the objective of improving the functional properties of these cellulosic materials and allowing their use in food containers which are suitable for high moisture and fats products. These technological advancements both promote reduction in single-use plastics and extend the shelf life of contained food, contributing to the food waste reduction [9].

In this context, it is essential to develop sustainable coatings that not only ensure the functionality of packaging materials but also safeguard ecosystems. The transition towards cellulosic materials with properties comparable to conventional plastics has generated growing interest within both the scientific community and industry, owing to their potential to reduce dependence on synthetic polymers and mitigate environmental impact. This bibliographic review provides a comprehensive overview of advances in packaging-based cellulosic substrates intended for food contact applications, with particular emphasis on their barrier performance against water and fats. Furthermore, it discusses the main technical challenges, cellulose modification strategies and application prospects, offering the reader a broad perspective on how these alternatives contribute to addressing the environmental and food safety challenges of the 21st century.

## 2. Food Loss and Waste

One of the main global problems in the food industry includes the huge economic losses that food waste generates during its production along with its impact on the environment, contributing to pollution. Despite not having a unique definition for this concept, the Food and Agriculture Organization of the United Nations (FAO) defines it as the reduction on quantity or quality along the food chain supply [10]. In 2022, The United Nations Environmental Programme (UNEP) estimated that 1050 million tons of food were wasted around the world, which corresponds to 132 kg per capita a year [4].

Food loss along the food chain supply can be related to multi factors such as weather conditions which damage crops, inadequate post-harvest practices and the mismatch between supply and demand, resulting in an excess in food production. In contrast, food loss that happens mainly during the final stages of food chain and represents around 60% of food waste is mostly originated by expired products, leftovers and excessive or impulsive purchases from buyers [11].

These large volumes of food waste have a significant impact on environmental sustainability, as they not only imply the loss of resources such as water, but also pollutant emissions that directly contribute to climate change. It is estimated that food loss and waste is responsible for around 14% of greenhouse gas (GHG) emissions worldwide, contributing to global warming. In particular, during the decomposition of organic waste in landfills, large volumes of methane (CH_4_) are generated, a gas that is 28 times more powerful than carbon dioxide (CO_2_) and remains in the environment for 12 years before decomposing in water vapor and CO_2_, which makes it a very noticeable dangerous pollutant [12].

Given this context, food packaging plays an essential role within the food chain supply, helping to reduce food loss and waste, extending the shelf life of products as well as protecting them from external agents or mechanic damage, thus contributing to food loss and waste reduction during production, distribution and sale [13].

## 3. Food Packaging

Food containers have a primary objective to protect the product during its shelf life from pathogen agents, chemical substances and physical damage during transport and storage [14,15]. In addition, they have to keep required conditions inside the packaging, slowing down the oxidative and degradative processes while preserving the organoleptic properties such as taste, color and texture [16,17].

In the food industry, the most used material for packaging manufacturing is traditional plastic derived from fuel due to its non-toxicity, low cost, high resistance and lightness [18]. However, these types of materials cause a global environmental problem because of their dependence on finite resources, accumulation and persistence in marine and land ecosystems due to inadequate waste management. Most of these plastics, having a high molecular weight, do not biodegrade easily and take decades or more to decompose in natural land. This is even slower in oceans due to the decrease in mechanic forces and photolysis, generating a serious problem in the ecosystem because they fragmentate into small particles of 5 mm, known as microplastics, which may be transferred along the food chain, severely impacting a great range of animals and consequently, humans [19,20]. It is estimated that people ingest on average between 0.1 and 5 g of microplastics weekly through different exposure routes, which leads to possible toxicological consequences on the gastrointestinal, respiratory, reproductive, immune, nervous and circulatory systems [21,22].

These problems are getting worse under an increasingly plastic industry, whose production reached 400.3 million tons in 2022, in which China led with 32%, followed by United States and Europe with 17% and 14%, respectively. From this production, between 40% and 50% is used for plastic packaging, half of them being single-use packaging that has been identified as the main cause of plastic contamination [19,23,24].

Given this problematic situation, the industry has recently focused on replacing plastic packaging derived from oil for more environmentally friendly containers from natural and renewable sources, which may mitigate the negative impact caused by traditional plastic material. Under this premise, research has focused on the study of biopolymers that can be defined as natural polymers derived from biological sources, that is to say, synthetized by animals, plants and microorganisms [25]. These plastics have become a viable alternative to polymers of fossil origin, having certain competitive advantages such as their high biodegradability, biocompatibility and non-toxicity [26].

Currently, poly(lactic acid) (PLA) and poly(hydroxyalkanoates) (PHAs) are two of the most studied and used biodegradable polymers by the industry, because they have similar properties to plastics derived from oil, making them a viable option to substitute conventional polymers established in the industry [27]. Nevertheless, producing these materials is costly, between USD 4000 and 15,000 per metric ton, which represents a significant difference compared to synthetic polymers, whose average cost is around USD 1500. This economic disparity places biopolymers at a competitive disadvantage, especially in industries that consider cost as a key factor [28,29].

Despite this fact, several studies and research have focused on optimizing processing technologies with the objective of reducing costs related to obtaining raw materials as well as biopolymers production. This trend responds to sustained growth projections demanding the origin of raw materials, triggered by an increase in public consciousness about the adoption of sustainable productive models, thereby fostering a circular economy based on the use of renewable resources and the reduction in residues [30].

## 4. Cellulose-Based Packaging

Nowadays, one of the most promising alternatives to replace plastics in the packaging industry is cellulosic materials made of paper or cardboard due to its competitive advantages compared to conventional materials already established in the industry. On the one hand, this type of packaging is associated with a lower carbon footprint, as renewable raw materials require less energy during both production and transportation. Moreover, being biodegradable, they avoid energy-intensive disposal processes such as incineration. On the other hand, cellulose-based packaging promotes a more efficient use of resources, since the incorporation of renewable feedstock and by-products from other production chains, such as agricultural residues, reduces reliance on non-renewable inputs [5,31].

Cellulose is the most abundant polymer in the world, being present in vegetables, fungi, algae, bacteria and animals. This polysaccharide is composed of a lineal array of anhydroglucose units (AGU) linked by C1 and C4 carbons through a *β*-1,4-O-glucosídic bond, constituting a repetitive disaccharide known as cellobiose [5]. Generally, the degree of polymerization (DP) of cellulose ranges between 200 and 10,000 [32].

This biopolymer organizes itself to produce elemental fibrils of around 30 to 36 glucan chains with a diameter which varies between 2 and 10 nm. These elements aggregate between them to form microfibrils that are considered the basic structural unit of cellulose. At the same time, microfibril nets, joining between them, form macrofibrils, which along with hemicellulose and lignin contribute adherence to the structure, forming cellulose fibers [33].

The main sources of extraction of cellulosic fiber are sugar cane, sisal fibers, wheat straw, coffee husk, banana peel, rice husk and wood pulp. In addition, depending on the raw material origin, cellulosic fibers show variable compositions regarding cellulose, hemicellulose and lignin, influencing its chemical and physical properties [34].

An important parameter that governs the performance of cellulose is its crystallinity index (CI), which reflects the relative proportion of crystalline to amorphous domains within the polymer. A high CI is associated with greater mechanical strength, stiffness and chemical resistance, since the ordered crystalline regions provide structural stability and reduce accessibility to solvents and reagents. Conversely, a lower CI implies higher amorphous content, which increases flexibility, water uptake and reactivity, but reduces barrier properties against gases and moisture. In packaging applications, controlling the CI is therefore critical: high crystallinity enhances durability and barrier efficiency, whereas moderate crystallinity can facilitate functionalization or chemical modification of the fibers. The crystallinity index is influenced by the cellulose source, extraction method and subsequent treatments, making it a decisive factor for tailoring cellulose-based substrates to specific requirements in sustainable packaging [35,36].

With regard to the selection of specific biomass sources, it is important to highlight their relative cellulose yield, purity and potential applications compared with other agricultural residues. For instance, wood pulp typically exhibits cellulose contents above 90%, with low lignin fractions, making it a highly pure source widely employed in the paper and packaging industries. In contrast, agricultural residues such as rice husk or wheat straw contain lower cellulose levels (30–45%) and higher proportions of lignin and hemicellulose, which require more intensive pretreatments to achieve comparable purity [34]. Sugarcane bagasse and sisal fibers are particularly attractive due to their relatively high cellulose content (50–70%) and availability as by-products of agro-industrial processes, thereby improving the economic feasibility of their use [32,34].

It is possible to obtain different degrees of separation between cellulose fibers through the mechanic and chemical treatment. For instance, microfibrillated cellulose (MFC) with a thickness between 10 and 100 nm is generated by means of homogenization at high pressure of the cellulosic fiber (CMF). Through the oxidation process by 2,2,6,6-tetramethylpiperidine-1-oxyl, commonly named TEMPO, nanofibrillated cellulose (NFC) with a diameter of 4 to 20 nm is obtained. Isolation of crystalline zones of cellulose through acid hydrolysis followed by a mechanic treatment generates nanocrystalline cellulose (NCC) that has a thickness raging between 5 and 70 nm [35,36]. A great quantity of structures can be generated from these compounds.

Cellulose-based materials are obtained through the fibers interlacing, generating structures that can be simply divided into flexible (paper) and rigid (cardboard, molded pulp, corrugated cardboard), according to their grammage. The drawback is that between fibers are empty spaces, generating porosity which causes low-barrier properties for gases and high absorption of water and fats, constituting a problem for food packaging [37,38,39]. To address this problem, the industry has the need to coat these materials with the purpose of generating barrier properties. In this respect, different polymeric coatings such as poly(propylene), poly(ethylene) and poly(ethylene terephthalate) have been used, increasing their resistance to water and fats. Nevertheless, the coated material is little degradable and difficult to recycle, affecting the process of circular economy [38,40,41]. In addition, coatings containing fluoride, such as perfluoride and polyfluoroalkyl substances (PFAS) have been used, remarkably improving their hydrophobicity and oleophobicity. Nonetheless, their use is related to toxicologic potential risk for human health and their use in food packaging has been gradually discontinued [42,43]. Considering the above, there is a need for researchers to focus their studies on the development of coatings that provide repellence to fats and humidity, keeping the material’s innocuity and biodegradability.

## 5. Biopolymeric Coatings

In recent times, new coating types (aliphatic polyesters such as poly(butylene) (PBS), poly(hydroxyalkanoates) (PHAs) and poly(lactic acid) (PLA)) that do not cause a negative impact on the environment have been developed. These have been considered as new alternatives to improve the properties of cellulosic materials such as paper and cardboard. Also, renewable polysaccharides such as starch, lignin, chitin and chitosan are of great interest, which show a big quantity of hydroxyl groups that provide a strong adherence and stability of cellulose fibers due to the formation of hydrogen bonds [9].

### 5.1. Poly(Lactic Acid) (PLA)

Poly(lactic acid) (PLA) is a semicrystalline polymer of natural origin, biodegradable and compostable, and mainly obtained from the fermentation of corn starch, sugar cane and cassava roots [44]. PLA synthesis is carried out from lactic acid, which coexists in its two stereoisomeric forms: *L*-lactic acid (levorotatory) and *D*-lactic acid (dextrorotatory). After a purification and polycondensation process, cyclic lactides in the forms *L*-lactide, *D*-lactide and meso-lactide are obtained, producing poly(lactic acid) by means of polycondensation or ring-opening polymerization (ROP) [45,46].

This biopolymer has been widely applied as a coating of cellulose packaging to increase its hydrophobicity. Wang et al. [47], who developed a study on active packaging made of modified cellulose coated with PLA for its application in shiitake fungi storage, successfully increased the contact angle with water from 11.42° to 132.12° and decrease water absorption of the material from 182.52% to 55.71%. In addition, PLA as a coating on a Kraft paper surface has been evaluated, obtaining a decrease in water absorption from 3 to 8.5 times the Kraft paper control (COBB 30), which decreased when the loading of the PLA coating increased. In addition, a gradual increase in the contact angle with water 15° above the angle of the uncoated paper was reported [48].

Also, PLA dispersions with PEG-PLA-PEG copolymer as a surfactant and xanthan gum as a thickener have been used to coat printing paper, reporting that water absorption of the substrate (COBB60) decreased according to the number of applied layers and the xanthan gum content, which reached values of 2.1 g/m^2^. In addition, a reduction in water vapor transmission rate (WVTR) values was obtained, with values of 60% and 75%, after applying coatings of 10 and 15 g/m^2^ of PLA with 0.8% of xanthan gum, respectively [49]. Likewise, Abdenour et al. [50] assessed the coating of PLA and xanthan gum on the paper and obtained a reduction in the WVTR, where the uncoated paper showed a value of 419 g/m^2^ d, decreasing to 35 g/m^2^ d, after applying a coating charge of 15 g/m^2^.

On one hand, it has been evaluated the use of coatings of PLA with nanoparticles of sync oxide (ZnO) on Kraft paper, which have accomplished a reduction around a 90% in the water absorption (COBB60). On the other hand, the contact angle of water decreased between 20° and 25°, according to the control substrate. These results show that whether the coating significantly improves resistance to water absorption, it can partially compromise superficial hydrophobicity of the substrate [51]. Additionally, foams of cellulose/chitosan have been coated with PLA, generating a hydrorepellent barrier on the material, achieving a reduction in water absorption around a 90%, as well as observing an increase in the contact angle from 84° for control, to 110° for the coated foam with a 25% of PLA [52]. Similarly, a group of researchers used a film of poly(lactic acid) (PLA), cellulose nanocrystals (CNC) and MnO_2_ to coat the paper sheet, reporting that WVTR decreased to 49% by coating the substrate with PLA until it reached 84%, when CNC/PLA was used with 3% of MnO_2_ in weight [53].

In conclusion, poly(lactic acid) (PLA) has demonstrated to be highly effective coating to improve the barrier properties of cellulosic substrates, significantly reducing water absorption (COBB) and water vapor transmission rate, which reflects a bigger superficial hydrophobicity. Additionally, the incorporation of additives such as xanthan gum, copolymers, sync oxide nanoparticles and cellulose nanocrystals has enhanced even more of these properties, adapting the performance of the coating according to material’s requirements and applicability, especially in the sustainable food containers field. Moreover, the use of PLA as a coating on cellulosic substrates, rather than manufacturing fully PLA-based packaging, represents a more economically feasible approach. In this configuration, the cellulosic substrate provides the bulk structure at low cost, while the PLA layer ensures the required barrier properties. This strategy reduces the overall material consumption of PLA, thereby lowering production costs while still achieving the functional and sustainability benefits associated with biopolymer-based packaging.

Table 1 shows a summary of the main results obtained on how different PLA coatings applied on cellulosic substrates perform as barriers to water.

### 5.2. Chitosan

Chitosan presents itself as the second most abundant polysaccharide on the planet after cellulose, which is mainly obtained from the exoskeletons of crustaceans. However, it can also be found in cell walls of insects, fungi and bacteria [34,54]. Its synthesis can be achieved from deacetylation of chitin, obtaining different molecular weights and different degrees of deacetylation [38]. Its application in food packaging becomes promising due to it showing antimicrobial activity and a good resistance to fats, as well as its being biodegradable and non-toxic [55].

Given this context, chitosan is a subject of research, used as coating on cellulosic material with the purpose of improving its resistance to fats. In some cases, it has been used in formulations with other compounds with the objective of providing repellence to humidity.

Kansal & Rabnawaz [56] carried out a study that analyzed the use of the chitosan coating grafted with sunflower oil on paper, reporting a reduction of 72% in water absorption, compare to paper without coating. Regarding the grease kit, a value of 8/12 for the coated paper was obtained. Grafted chitosan with poly(dimethylsiloxane) on paper has also been investigated, obtaining a repellence to fats of 8/12 in the substrate with the grafted polymer. In the case of resistance to water, (COBB60), the grafted coating decreased to 21 g/m^2^, compared to 35 g/m^2^ of paper with no coating [57]. Another study focused on evaluating the effect of coated paper with a grafted chitosan solution with castor oil to improve its resistance to water, reporting a contact angle with water of 92.9° initially and 85.46° at 1400 s, compared to 79.36° and 38.23° of paper with no coating in the same time period. Similarly, this coating increased the contact angle by 35% with oil (30.12°), compared to coating chitosan without additives (19.61°), reaching a contact angle similar to paper with no coating (29.96°). On WVTR, chitosan coating grafted with oil decreased by around 50% (50% R.H.) and 80% (0% R.H.) [58]. These results indicate that grafting different compounds to chitosan noticeably improves resistance to water and fats.

Studies have also been conducted using, as coating, a mix of chitosan/montmorillonite on Kraft paper. The result obtained showed that the coated material with 2% (*w*/*v*) of chitosan and 0.1% (*w*/*v*) of montmorillonite reached a grease kit of 9/12 over 7/12 and 8/12 of chitosan coatings with 2.5% and 3% (*w*/*v*), respectively, which could be related to the synergic action between chitosan and nanoclay, reinforcing the coating matrix, as well as reducing lipid permeability [59]. In another study, paper was coated with an emulsion of chitosan and carnauba oil, where the coating with 3% of chitosan and 90% of carnauba oil, regarding total solids, exhibited a high water-repellent and oleophobic barrier, having a water absorption (COBB60), a contact angle of water and a grease kit of 7.5 g/m^2^, 130.9° and 12/12, respectively [39]. On the other hand, Shi et al. [60] assessed the use of a chitosan coating, genipin and microfibrillated cellulose on printing paper of 70 g/m^2^ and 80 g/m^2^ grammage, observing that after using a composition of 2% by weight of chitosan, 0.1% of genipin and 15% of microfibrillated cellulose, a bigger resistance to fats was obtained for a kit value of 12. In addition, they reported that WVTR decreased between 22% and 28% with respect to paper controls.

Thus, chitosan has demonstrated to be a versatile and effective material as coating for cellulosic substrates, especially improving resistance to fats and humidity. Its combination with compounds such as vegetable oil, poly(dimethylsiloxane) and montmorillonite, among others, enhances its barrier properties, allowing it to reach remarkable results in tests for the grease kit, water absorption, contact angle and a water vapor transmission rate, placing it as a good alternative for the development of coatings for packaging in contact with food.

Table 2 presents the main results obtained regarding resistance to water and fats of cellulosic substrates coated with chitosan and its modified formulations.

### 5.3. Lignin

Lignin is one of the most abundant phenolic structure biopolymers in the planet, which can be found in cellulose, joining its fibers with hemicellulose to provide a better resistance to cell walls [61]. This polymer is mainly composed by three different monomers: coniferyl alcohol (G), sinapyl alcohol (S) and *p*-coumaryl alcohol (H), where its proportion varies according to the vegetal source [62]. This compound is mainly produced in the paper industry where it is obtained as a subproduct of processes and used as low-value fuel to generate electricity and heat [63,64]. Thus, every year the pulp and paper industry generates approximately 70 million tons of lignin, and just 2% is allocated to manufacture commercial products such as dispersants, surfactants and additives [65,66].

Several studies have reported that the incorporation of lignin as an additive in the paper production processes may contribute to the resistance to water because this compound shows nonpolar functional groups, providing hydrophobicity to the material. Shorey & Mekonnen [67] carried out research that studied the effect of esterified lignin in a PBAT solution used as coating on paper, reporting that the incorporation of this compound of 50% by weight in the varnish increased the contact angle with water by 72.6%, compared to pure PBAT coating. In addition, from COBB120, the esterified lignin addition of 50% by weight decreased the water absorption by 40%. For resistance to fats, this increased to 5/12 with respect to the value of 1/12 of PBAT without lignin. These results show that the incorporation of esterified lignin remarkably improves the hydrophobic and oleophobic properties of the PBAT coating on paper. Moreover, the effect of a lignin and cationic starch nanoparticle coating on A4 cellulose paper has been evaluated, showing that it increases the contact angle from 104°, for control, to 118° for coated paper. Water absorption (COBB60) noticeably decreased with the incorporation of the coating. Similarly, WVTR decreased from 2569 g/m^2^ d for the sample with no coating, to 426 g/m^2^ d for the sample coated with lignin/cationic starch. In addition, grease resistance increased the kit from 1/12, for paper, to 9/12 for coated paper [68].

On the other hand, cellulose and cellulose/lignin films have been developed, reporting that the incorporation of lignin decreased water absorption to 40.8% of humidity content, compared to 75% of the cellulose film [69]. Likewise, the effect of a poly(vinyl acetate)/lignin copolymer coating was studied to be used on paper, reporting that the PVAc coating on the substrate increased the contact angle with water from 2.1°, for control paper, to 80° for coating. When lignin was incorporated, PVAc resistance to water improved even more, recording a contact angle of 90° [70]. In another work, an esterified lignin and cellulose acetate coating was studied to be applied on a paper sheet manufactured from Miscanthus giganteus cellulose, obtaining a contact angle with water of 134°, compared to 52° for uncoated paper [66].

Resistance to water that generates a lignin coating with cellulose on filter paper has been analyzed, using xylan as a compatibilizer, obtaining, as a result, a reduction to 83%, compared to absorption of 220% for untreated paper. Similarly, WVTR showed the same behavior, reducing its permeability by 74%, compared to substrate control. In contrast, by analyzing the contact angle with water of the filter paper, immediate adsorption of the droplet was observed, which is related to its high porosity, preventing its angle from being measured. Nevertheless, after coating the material, it showed a contact angle of 81°, demonstrating the paper water repellence [71]. Finally, Xie et al. [72] developed a modified lignin coating (sodium lignosulfate) using cellulose nanocrystals and poly(vinyl alcohol) as a crosslinker on paper; having a ratio of 4:1 of lignosulfate and cellulose nanocrystals showed the best behavior, decreasing water absorption (COBB30) to 15%.

In conclusion, lignin has demonstrated great potential as an additive and functional component of coatings for cellulosic substrates, greatly improving its barrier properties to water and fats. Its incorporation, naturally or modified, has allowed the increase of its water repellence and oleophobicity. These results promote the development of more sustainable and effective container materials for the food industry.

Table 3 shows a summary of the main results obtained regarding resistance to water and fats of coated lignin cellulosic materials.

### 5.4. Cationic Starch

Starch is a polymer composed of two polysaccharides: amylose and amylopectin, found in a proportion of 20–25% and 75–80%, respectively. Amylose has a long lineal structure linked by *α*(1→4) bonds, while amylopectin has a branched structure linked by *α*(1→4) and *α*(1→6) bonds [73]. Thanks to its high availability, low cost and biodegradability, amylose represents a material that is extremely interesting to the industry. However, its application in the form of films is limited by its hydrophilic nature and deficient mechanic and thermal properties [74]. For this reason, it is necessary to make chemical and physical modifications to this polymer, thus adjusting the desired functionality according to each industrial field.

Cationic starch is a chemically modified starch with cationizing reagents such as 2-diethylaminoethyl chloride and 2,3-epoxypropyltrimethylammonium chloride, generating a reaction of etherification in native starch [75,76]. This way, starch properties such as viscosity, solubility, crystallinity and biodegradability are modified thanks to the present cationic groups. This polysaccharide, having several hydroxyl groups on its surface, shows similar characteristics to cellulose and hemicellulose of paper, which produces a great affinity to cellulosic materials. Consequently, this material has been extensively used as coating for the cellulose industry with the objective of improving the smoothness of the paper and increasing the sharpness of subsequent prints, by mixing it with different reagents, therefore providing resistance to water and fats [77].

A study analyzed the behavior of Kraftliner paper after coating it with a cationic starch suspension, observing that its resistance to water decreased, reflecting a reduction of 13% in the contact angle and increased water absorption by 410%, with respect to uncoated paper. While performing the oil absorption test, this coating reached a value of 10/12, compared to 0/12 of substrate control. On the other hand, when incorporating 75% carnauba wax by weight of suspension, this increased the contact angle with water by 11% and water absorption by 51% with respect to uncoated paper. For resistance to oil, this increased kit values between 5 and 9/12, according to the wax concentration on the varnish [73]. Another study evaluated the effect of a cellulosic nanocrystalline coating and cationic starch on Kraft papers, reporting a reduction in water absorption when the material is coated with a solution of 5% of nanocrystalline cellulose and 95% of cationic starch, going from 65.43 g/m^2^, for uncoated paper, to 33.28 g/m^2^ for coated paper, as a result of the strong hydrogen bonds that are produced between hydroxyl groups of starch with nanoparticles. After performing an oil resistance test, it was reported that aggregating this coating reduced permeability to fatty compounds by 60% [76].

Furthermore, alkyl ketene dimer (AKD) and cationic starch have been applied to paper, by means of a wet-end and surface chemical addition, observing that this coating could decrease water absorption to between 82% and 92% in both cases. Similarly, this treatment increased resistance to fats to values as far as 2–3/12 [78]. In addition, in the case of cardboard coating with cationic starch of high and low molecular weight, it was reported that they caused a decrease by 10% in permeability to water vapor, with respect to control, having no differences between the two types of starch. On resistance to fats measured by kit, cationic starch of high and low molecular weight increased its value as far as 7/12 and 2/12, respectively, compared to 0/12 of uncoated cardboard. Additionally, water and oil absorption were measured, by means of COBB60 test, observing that coating with cationic starch, independent of molecular weight, produced an increase in water absorption, due to the hydrophilic character of the compound. In contrast, oil absorption decreased with the cationic coating, due to starch’s high molecular weight which had the best performance, because has better properties of formation of a homogeneous and defect-free film [79].

Given these results, cationic starch is considered an effective coating to improve the resistance to fats in cellulosic substrates, due to the fact that its hydrophilic nature may increase water absorption. Nevertheless, its performance can be optimized through the incorporation of additives such as waxes, nanocellulose and alkyl ketene dimer among others, thus improving barrier properties against water.

Table 4 shows a summary of the main results of barrier properties of cationic starch-based coatings.

### 5.5. Whey Protein Isolate (WPI)

Proteins comprise a wide range of biopolymers present in animals and plants. These are composed of long chains of various amino acids linked together, providing many characteristics. They are organized into globular structures and fibrous structures, depending on the interactions and bonds that occur between the same amino acids [80,81].

Whey protein isolate is obtained as a subproduct during cheese making and is composed of *β*-lactoglobulin, *α*-lactalbumin, bovine serum albumin, immunoglobulins and specific polypeptides [82]. According to the degree of purity of this protein, it is divided into whey protein concentrate (WPC) and whey protein isolate (WPI), where the latter goes through an additional purification process, providing a higher concentration of proteins. Whey protein has an excellent capacity for forming films that act as barrier against oxygen, aroma and oils. Because of its functionality, versatility, biodegradability and interaction with other compounds, its application is widely being studied in the food packaging field, with the purpose of replacing traditional plastic packaging [83].

Although the study of whey protein isolate (WPI) on cellulosic material is limited, its use as a base for film and coating formations on other polymers has been investigated. Accordingly, Song et al. [82] studied the effect of WPI as an intermediate layer between PET and LLDPE films for its application in fried rice with shrimp packaging, reporting that the film with WPI exhibited greater performance as a barrier to oxygen and water vapor with values of 51.7 cm^3^ μm/m^2^ d atm and 7.1 g μm/m^2^ d kPa, respectively. Another study of WPI films was developed, with galactooligosaccharides (GOS) and xylooligosaccharides (XOS) as additives, with the objective of assessing their effect on the material. Aggregation of these prebiotics decreased permeability to water vapor of the WPI film from 1.09 g mm/h m^2^ kPa to 0.63 g mm/h m^2^ kPa, showing no statistical differences between the prebiotic and its concentration. In contrast, the incorporation of these additives decreased the contact angle with water, compared to film without prebiotic. Given this context, these films may be used as semipermeable barriers for food, where condensation of water vapor is not desirable [84]. In addition, the use of a coated film of PET with WPI and montmorillonite nanoplatelets (MMT) was evaluated, reporting that the aggregation of this compound decreases the protein hydrophilic nature, reducing permeability to water vapor as far as 60%, with respect to the coated films only with WPI [85]. Another study, after evaluating whey protein isolate (WPI) films with transglutaminase (TG) and sunflower oil, observed that transglutaminase decreased the humidity absorption by 23%, compared to the WPI film. This effect decreased as far as 42% with the incorporation of 3% sunflower oil by weight. On permeability to water vapor, a similar behavior was reported, where it decreased from 1.38 g mm/m^2^ h kPa, for WPI, to 0.48 g mm/m^2^ h kPa for the WPI film with TG and sunflower oil by 1.5%, by weight. This behavior is based on controlled incorporation of sunflower oil in WPI emulsions, crosslinked with TG, improving structure and resistance to water of the films [86].

In conclusion, whey protein isolate (WPI) is an outstanding additive to develop biodegradable coatings and films, due to its capacity for forming effective barriers against humidity and fatty compounds. Although its use on cellulosic substrates is limited, several studies have demonstrated that its functionality may greatly improve by incorporating other additives such as prebiotics, montmotillonite and transglutaminase (among others), adjusting its permeability and hydrophobicity properties.

Table 5 shows the main results for the hydrophobicity tests of different materials with WPI in its structure.

### 5.6. Sodium Alginate

Sodium alginate is a biopolymer that is found naturally in brown algae and composed of *β-D*-mannuronic acid (M) and *α-L*-guluronic acid (G), which are linked in linear configuration, by (1→4) glucoside bonds [38]. It is widely studied in both food and packaging industries, due to its great capacity for forming films, its biocompatibility, non-toxicity, biodegradability and abundance. Given these characteristics, several studies have focused on its use as an ecological matrix for coating of edible food, coatings of polymeric containers or as packaging films [87]. Nonetheless, its use is limited due to its deficient barrier properties against water and UV light, apart from its low antimicrobial and antioxidant capacity [88]. One way of modifying the inherent properties of sodium alginate consists of aggregating it with other compounds that act as plasticizers or supplements that incorporate new characteristics or improve native properties.

Kopacic et al. [89] carried out a study where a surface of a paper made from primary and secondary fibers was coated with sodium alginate in order to compare its properties with the chitosan coating on the same paper. The results demonstrated that the alginate coating reduced the water vapor transmission rate (WVTR) by 44%. On one hand, COBB60 results showed an increase of 116%, while the contact angle decreased to 35°, compared to ~90° of control. These results may indicate that alginate caused a hydrophobic effect on the substrate. On the other hand, the application of this compound noticeably improved resistance to fats, increasing the kit value from 0/12 for substrate control, to 12/12 for coated paper.

Another study analyzed the effect of a coating of sodium alginate and oxidized nanocellulose/silver nanoparticles paper sheets, observing a reduction in permeability to water vapor (WVP) as far as 25%, from a WVP of 5.44·10^−7^ g/s m Pa, for uncoated paper, to 4.08·10^−7^ g/s m Pa for a sheet coated with sodium alginate [90]. Priyadarshi et al. [91] assessed the behavior of coatings of sodium alginate on Kraft paper sheets, reporting that the coated paper with alginate decreased water absorption by 3%, compared to the uncoated paper. Similarly, oil absorption showed a reduction of around 33% with the coating on the substrate. On WVP, the incorporation of alginate slightly increased permeation, showing no statistical differences with material control. Another research characterized alginate, chitosan, EVOH and microfibrillated cellulose to evaluate its application as coating on paper and cardboard, reporting that alginate showed a high resistance to fats reaching kit values of 12/12; therefore, it would have potential to be used as container of fatty products [92].

In summary, sodium alginate has demonstrated to be an effective coating to improve resistance to fats on cellulose-based substrates, even though its application seems to be limited because of its high affinity with water. However, its high formation of homogenous films and compatibility with other compounds make it a versatile candidate to develop food containers that require a lipid barrier.

Table 6 shows a summary of the main results of studies mentioned before.

### 5.7. Waxes

Waxes are long aliphatic hydrocarbon chains of between 10 and 60 carbon atoms, such as alcohols, ketones, aldehydes, esters and fatty acids that do not have polarity in their molecular structure; that is to say, they are nonpolar molecules [93]. Waxes are classified into natural and synthetic, where the first are subdivided into renewable natural and non-renewable natural waxes. Renewable natural waxes come from vegetable sources such as carnauba wax, candelilla wax, beeswax and wool wax (lanolin). These can be modified through chemical processes such as hydrogenation and re-esterification. Non-renewable natural waxes come from minerals such as lignite [94]. In contrast, synthetic waxes come from petrochemical compounds which are subdivided into paraffin waxes (macrocrystalline) and microcrystalline waxes. The first are produced from the distillation of petroleum under pressure and heat, forming unbranched hydrocarbons with a linear structure and hard, brittle consistency chains. Microcrystalline waxes are produced from dewaxing processes for lubricating oil waste, generating unbranched and cyclic hydrocarbons whose crystals are smaller, providing greater flexibility and plasticity [95].

Given their high availability, economic viability, excellent hydrophobic properties and high compatibility with polysaccharides and proteins to form composite films, they have attracted attention from researchers to be used as a material for food packaging which requires resistance to water [93,96].

Liu et al. [97] evaluated a coating of microcrystalline wax on paper previously coated with cationic starch to analyze its resistance to water and oil, observing better behavior when the load of coating on paper was 10 g/m^2^, reaching a classification kit value of 12/12, contact angles with the advancing and receding oil of 72.1° and 50.4°, respectively, a COBB60 of 4 g/m^2^ and contact angles with the advancing and receding water of 126.5° and 96.7°, respectively. The permeability values to water vapor (WVP) decreased by 96.7%, with respect to uncoated paper. In another study, a double coating of cationic starch and carnauba wax on Krafliner paper was applied, where it was observed that a mix of 25% of cationic starch and 75% of carnauba wax increased the contact angle with water from approximately 90°, for control, to 100° in the coated substrate. On the other hand, water absorption measured by means of COBB120 test increased by 51%, which was associated with paper porosity and a larger amount of hydroxyl groups present in cationic starch, promoting the swelling of the coating. When resistance to fat was analyzed, a classification kit value of 5/12 was obtained, compared to 0/12 of material control [73].

Another research assessed the behavior of Kraft paper coated with chitosan and vegetable wax extracted from banana leaves in different compositions (0%, 1%, 3%, 5% and 7% by weight), where the uncoated paper showed a contact angle with water of 65.1° that increased as far as 123.2° after coating it with 3% vegetable wax. Water absorption of coated samples with wax decreased to between 20% and 80%, with respect to substrate control. On the percentage of oil sorption, the only coatings that showed a reduction were 3% and 5% vegetable wax, which was associated with a bigger concentration of chitosan as a protective barrier against oil [98].

A study was also carried out using mixtures of bee wax and shellac wax as coatings on filter paper, in proportions of 100:0, 75:25, 50:50, 25:75 and 0:100, where contact angles with water that varied between 134° and 147° were observed, providing a surface with high hydrophobicity. In addition, water absorption of filter paper was measured after 15 min in contact with water, where the uncoated sample absorbed 12.29% of water, while coated samples adsorbed only 0.64% [99].

Researchers Lee and Lim [100] prepared coatings of paraffin wax, polyolefin wax, and a mixture of both on white fine paper, reporting that the coated paper with polyolefin wax showed a lower WVP value, corresponding to 29.4 g/m^2^ d. On the contact angle with water on coated surfaces with paraffin wax, a mixture of it and polyolefin wax exhibited similar behavior between them, with a contact angle of approximately 100° at time 0 which gradually decays to 70° after 10 h, which is above the 75° and 45° at time 0 and 5 h of contact for the uncoated paper, respectively. In another study, Zhang et al. [39] obtained a photocopy paper with a superhydrophobic surface through the application of a mix of bee and carnauba waxes, where they reported that the proportion of 5:5 of bee and carnauba waxes increased the contact angle with water by 57° with respect to the uncoated paper, reaching 161.7°, and indicating that the emulsion reduced the paper superficial energy, therefore successfully creating a highly resistant surface to water.

Given their apolar structure and compositional versatility, waxes have demonstrated to be effective additives as coatings for cellulosic packaging, improving water and fat repellence. Their single or combined incorporation with other polymers enhances their attractive properties even more in applications of food packaging.

Table 7 exhibits the results of barrier properties to water and oil of different coatings based on natural waxes on cellulosic material.

## 6. Environmental Impact

With regard to the quantification of the environmental impact associated with cellulosic substrates coated with natural compounds, the task is complex, mainly due to the wide variability of raw materials employed as barrier-forming agents against moisture and fats, as well as differences in the methods of extraction and application. In general terms, the carbon footprint of the pulp and paper industry has been estimated to lie within an approximate range of 0.4 to 0.5 kg CO_2_-eq/kg, depending on the type of product and the system boundaries considered [101]. Nevertheless, the incorporation of a natural coating onto the substrate inevitably increases the environmental impact, as it introduces additional emissions related to the synthesis or extraction of the raw material, the energy required for its processing and transport, and the mass load of the applied coating. It is noteworthy that, although this increase may be relatively modest when thin layers of renewable origin (e.g., chitosan, lignin and proteins) are employed, the impact can become more significant when production processes are energy-intensive or rely on chemical treatments. In this context, the choice of raw material, the energy efficiency of the coating stage and the thickness of the applied layer emerge as critical variables for minimizing the carbon footprint of the final material intended for food contact applications.

## 7. Conclusions

Food containers made from cellulosic materials have gained great recognition in recent years, driven by the growing awareness of society regarding the environmental impact that plastic derived from petrochemical sources generate. In this context, paper and cardboard containers have become the most important alternatives to replace polymeric materials of fossil origin. Despite their environmental advantages, they show certain limitations compared to their plastic counterparts. As main disadvantages, their high porosity related to cellulosic materials leads to a low barrier capacity to gases, as well as high water and fat absorption, making their application in packaging of certain foods difficult.

Overcoming limitations and preserving the functional properties necessary for food packaging have become two crucial objectives. Various coatings based on biopolymers have been studied and developed, whose characteristics include providing high resistance to humidity, oils and fats, as well as meeting biodegradability and compostability criteria, thus reducing the environmental impact associated with plastic residues. Poly(lactic acid) (PLA), cationic starch, chitosan, sodium alginate and different waxes, which provide improved barrier properties to water and fats in cellulosic-based material, are among the investigated biopolymers.

According to the evidence gathered through this bibliographic revision, these biopolymeric alternatives represent sustainable environmental solutions that provide new functional properties to cellulosic-based containers, owing to their suitable use in direct contact with foods rich in fats and water. Finally, this has been considered a significant step towards the development of solutions which are more responsible and aligned with current sustainability demands.

## Figures and Tables

**Table 1:** Barrier properties of cellulosic substrates coated with poly(lactic acid) (PLA).

Substrate	Coating	Control	Main Results	Reference
Modified Cellulose Sheet	PLA	WCA: 11.42° WA: 182.52% WVP: 1.78 × 10^−10^ g/Pa s m	WCA: 132.12–133.23° WA: 55.71% WVP: 4.22–4.25 × 10^−11^ g/Pa s m	[47]
Kraft Paper	PLA	Cobb30: 25.6 g/m^2^ WCA: ~65 g/m^2^	Cobb30: 3.2–8.8 g/m^2^ WCA: ~70–80 g/m^2^	[48]
Paper	PEG-PLA-PEG	Cobb60: 100 g/m^2^ WVP: 22 × 10^−6^ g/Pa d m	Cobb60: 2.0–50.0 g/m^2^ WVP: 6.7–9.7 × 10^−6^ g/Pa d m	[49]
Paper	PLA-Xanthan gum	WVP: 14.5 × 10^−6^ g/Pa d m	WVP: 1.5–3.8 × 10^−6^ g/Pa d m	[50]
Kraft Paper	PLA-ZnO	Cobb60: 24.2 g/m^2^ WCA: 96.1°	Cobb60: 1.8–3.4 g/m^2^ WCA: 71.2–76.1°	[51]
Cellulose/Chitosan foam	PLA	WCA: 84° WA: ~4 g water/g dry foam	WCA: 94–110° WA: 0.33–0.52 g water/g dry foam	[52]
Paper	CNC/PLA@MnO_2_	WVTR: 890.34 g/m^2^ d	WVTR: 450.65–145.46 g/m^2^ d	[53]

WCA: Water contact angle; WA: Water absorption; WVP: Water vapor permeability; WVTR: Water vapor transmission rate; Cobb: Cobb test.

**Table 2:** Barrier properties of cellulosic substrates coated with chitosan.

Substrate	Coating	Control	Main Results	References
Paper	Chitosan-graft Sunflower Oil	Cobb60: ~100 g/m^2^ WVP: 90.4 g μm/Pa d m^2^ WCA: 53.5° KIT: 0/12 OCA: <23°	Cobb60: ~30–60 g/m^2^ WVP: 53.4–69.2 g μm/Pa d m^2^ WCA: 94.3–98.3° KIT: 6–8/12 OCA: 53.1–56.9°	[56]
Kraft Paper	Chitosan-graft poly(dimethylsiloxane)/Zein	Cobb60: ~35 g/m^2^ WCA: 71.5° WVTR: 1200 g/m^2^ d KIT: 0/12 OCA: 40°	Cobb60: ~21–25 g/m^2^ WCA: 100–121° WVTR: ~122–1000 g/m^2^ d KIT: 8–11/12 OCA: 64–68°	[57]
Kraft Paper	Chitosan-graft Castor Oil	WCA: 79.36° WVTR: ~1500 g/m^2^ d OCA: 29.96°	WCA: 92.91° WVTR: ~300–800 g/m^2^ d OCA: 30.12°	[58]
Kraft Paper	Chitosan–Montmorillonite	WCA: ~110° KIT: 0/12 OCA: ~30°	WCA: ~90–100° KIT: 6–9/12 OCA: ~30–40°	[59]
Paper	Chitosan/Carnauba Wax	Cobb60: 22.8 g/m^2^ WCA: - KIT: -	Cobb60: 7.5–30.3 g/m^2^ WCA: >105° KIT: 9–12/12	[39]
Paper	Chitosan–Genipin–Microfibrillated cellulose	WVTR: ~1300–1450 g/m^2^ d KIT: 0–10/12	WVTR: ~900–1100 g/m^2^ d KIT: 10–12/12	[60]

WCA: Water contact angle; WVP: Water vapor permeability; WVTR: Water vapor transmission rate; Cobb: Cobb test; KIT: Kit test; OCA: Oil contact angle; -: Not Reported.

**Table 3:** Barrier properties of cellulosic substrates coated with lignin.

Substrate	Coating	Control	Main Results	Reference
Paper	Lignin–PBAT	Cobb60: 35.82 g/m^2^ WCA: 77.1° Cobb60 (Oil): 155.83 g/m^2^	Cobb60: 18.05–27.27 g/m^2^ WCA: 81.7–134.2° Cobb60 (Oil): 54.33–103.53 g/m^2^	[67]
Paper	Lignin–Cationic Starch	Cobb60: ~48 g/m^2^ WCA: 104° WVTR: 2569 g/m^2^ d KIT: 1/12	Cobb60: ~36–38° g/m^2^ WCA: ~100–118° WVTR: 426–864 g/m^2^ d KIT: 8–9/12	[68]
Cellulose/Lignin/Xylan Film	-	WA: ~75% WRV: 37.82%	WA: ~60% WRV: ~30%	[69]
Paper	Poly(vinyl Acetate)/Lignin	WCA: 2.1°	WCA: 80–90°	[70]
Paper	Esterified Lignin–Cellulose acetate	WCA: 52.4° WVTR: 213.7 g/m^2^ d	WCA: 132.6–133.7° WVTR: 63.3–64.2 g/m^2^ d	[66]
Paper	Lignin–Cellulose–Xylan	WA: 220% WVTR: 670.14 g/cm^2^ d	WA: 83% WVTR: 172.96 g/cm^2^ d	[71]
Paper	Sodium lignosulfonate–Cellulose Nanocrystals–poly(vinyl alcohol)	Cobb30: 66 g/m^2^	Cobb30: 56 g/m^2^	[72]

WCA: Water contact angle; WA: Water absorption; WVTR: Water vapor transmission rate; Cobb: Cobb test; WRV: Water retention value; KIT: Kit test.

**Table 4:** Barrier properties of cellulosic substrates coated with cationic starch.

Substrate	Coating	Control	Main Results	Reference
Kraft Paper	Cationic Starch–Carnauba wax	Cobb120: 37 g/m^2^ WCA: ~88° WVP: ~30 g mm/kPa m^2^ d KIT: 0	Cobb120: 56–73 g/m^2^ WCA: ~85–100° WVP: ~28–33 g mm/kPa m^2^ d KIT: 5–9/12	[73]
Paper	Cationic Starch–Cellulose Nanocrystals	Cobb60: 65.43 g/m^2^ OP: 88.14%	Cobb60: 33.28–43.24 g/m^2^ OP: 44.26–53.36%	[76]
Paper	Cationic Starch–AKD	Cobb60: ~140 g/m^2^ KIT: 1/12	Cobb60: ~10–25 g/m^2^ KIT: 3–4/12	[78]
Paperboard	Cationic Starch (high and low molecular weight)	Cobb60: 38.9 g/m^2^ WVP: 1.5 × 10^−9^ g m/m^2^ s Pa KIT: 0/12 OA: 25 g/m^2^	Cobb60: ~20–65 g/m^2^ WVP: ~1.3 × 10^−9^ g m/m^2^ s Pa KIT: 1–12/12 OA: ~2–18 g/m^2^	[79]

WCA: Water contact angle; WVP: Water vapor permeability; Cobb: Cobb test; KIT: Kit test; OA: Oil absorption; OP: Oil permeability.

**Table 5:** Barrier properties of cellulosic substrates coated with WPI.

Substrate	Coating	Control	Main Results	Reference
PET/LLDPE	WPI	WVP: 1566.21 g μm/m^2^ d Pa	WVP: 7.10 g μm/m^2^ d Pa	[82]
WPI/galactooligosaccharides WPI/xylooligosaccharides	-	WVP: 1.09 g mm/m^2^ h kPa WCA: 56.61°	WVP: 0.63–0.78 g mm/m^2^ h kPa WCA: 46.69–51.66°	[84]
PET	WPI-MMT	WVTR: ~1268–1539 g/m^2^ d	WVTR: 642–1323 g/m^2^ d	[85]
WPI-Transglutaminase-Sunflower oil	-	WVP: 1.38 g mm/m^2^ h kPa WA: 4.87%	WVP: 0.48–0.59 g mm/m^2^ h kPa WA: 3.70–3.95%	[86]

WCA: Water contact angle; WA: Water absorption; WVP: Water vapor permeability; WVTR: Water vapor transmission rate.

**Table 6:** Barrier properties of cellulosic substrates coated with sodium alginate.

Substrate	Coating	Control	Main Results	Reference
Paper	Alginate and Chitosan	WVTR: 609–690 g/m^2^ d WCA: ~100° Cobb60: 25–155 g/m^2^ KIT: 0–1/12	WVTR: ~200–400 g/m^2^ d WCA: ~70–100° Cobb60: 29–149 g/m^2^ KIT: 6–12/12	[89]
Paper	Alginate/oxidized nanocellulose–silver nanoparticles	WVP: 5.44 × 10^−7^ g/s m Pa	WVP: 4.38–4.88 × 10^−7^ g/s m Pa	[90]
Kraft Paper	Alginate–Sulfur quantum dots	WVP: 1.4 × 10^−9^ gm/m^2^ s Pa WA: 21% OA: 48%	WVP: 1.5–1.7 × 10^−9^ gm/m^2^ s Pa WA: 18–47% OA: ~15%	[91]
Sodium Alginate	-	-	WVTR: 258.95 g/m^2^ d WCA: ~65.2–72.2° KIT: 12/12	[92]

WCA: Water contact angle; WA: Water absorption; WVP: Water vapor permeability; WVTR: Water vapor transmission rate; Cobb: Cobb test; KIT: Kit test; OA: Oil absorption; -: Not Reported.

**Table 7:** Barrier properties of cellulose substrates coated with waxes.

Substrate	Coating	Control	Main Results	Reference
Paper	Microcrystalline wax	Cobb60: 23 g/m^2^ WVP: 5.436 × 10^−12^ g cm/cm^2^ s Pa WCA: 80.9° KIT: 0/12 OCA: 47.8°	Cobb60: 20.4–10.5 g/m^2^ WVP: 2.497 × 10^−12^–1.816 × 10^−13^ g cm/cm^2^ s Pa WCA: ~90–106° KIT: 1–8/12 OCA: ~55–68.4°	[97]
Kraft Paper	Cationic Starch–Carnauba wax	Cobb120: 37 g/m^2^ WCA: ~88° WVP: ~30 g mm/kPa m^2^ d KIT: 0	Cobb120: 56–73 g/m^2^ WCA: ~85–100° WVP: ~28–33 g mm/kPa m^2^ d KIT: 5–9/12	[73]
Kraft Paper	Chitosan–Plant-based wax	WCA: 65.1° WA: 147.9% OCA: 29° OA: 148.2%	WCA: 108.2–123.2° WA: 67.5–120.7% OCA: ~32° OA: 97.7–212.2%	[98]
Paper	Beeswax and Shellac wax	WCA: - WA: 12.19%	WCA: 134–147° WA: 0.64%	[99]
Paper	Paraffin wax–Polyolefin wax	WVTR: 2564.6 g/m^2^ d WCA: ~75°	WVTR: ~29–50 g/m^2^ d WCA: 90–100°	[100]
Paper	Chitosan–Carnauba wax	Cobb60: 22.8 g/m^2^ WCA: - KIT: -	Cobb60: 7.5–30.3 g/m^2^ WCA: >105° KIT: 9–12/12	[39]

WCA: Water contact angle; WA: Water absorption; WVP: Water vapor permeability; WVTR: Water vapor transmission rate; Cobb: Cobb test; KIT: Kit test; OCA: Oil contact angle; OA: Oil absorption; -: Not Reported.

## Data Availability

No new data were created or analyzed in this study.

## Abbreviations

The following abbreviations are used in this manuscript:

UNEP	United Nations Environmental Programme
FAO	Food and Agriculture Organization
PLA	Poly(lactic acid)
PHA	Poly(hydroxyalkanoate)
MFC	Microfibrillated cellulose
PEG	Poly(ethyleneglicol)
ZnO	Zinc oxide
MnO2	Manganese dioxide
PBAT	Poly(butylene adipate-co-terephthalate)
AKD	Alkyl ketene dymer
WPI	Whey protein isolate
MMT	Montmorillonite
PET	Poly(ethylene terephthalate)
LLDPE	Linear low-density poly(ethylene)
GOS	Galactooligosaccharide
XOS	Xylooligosaccharide
WCA	Water contact angle
WA	Water absorption
WRV	Water retention value
WVP	Water vapor permeability
WVTR	Water vapor transmission rate
OCA	Oil contact angle
OA	Oil absorption
OP	Oil permeability
